# Optimal Control of False Information Clarification System under Major Emergencies Based on Differential Game Theory

**DOI:** 10.1155/2022/7291735

**Published:** 2022-09-23

**Authors:** Bowen Li, Hua Li, Qiubai Sun, Rongjian Lv

**Affiliations:** ^1^School of Electronic and Information Engineering, University of Science and Technology Liaoning, Anshan, China; ^2^School of Business Administration, University of Science and Technology Liaoning, Anshan, China

## Abstract

To further study the issue of false information classification on social platforms after major emergencies, this study regards opinion leaders and Internet users as a false-information classification system and constructs three differential game models of decentralized, centralized, and subsidized decision-making based on optimal control and differential game theory. Comparison analyses and numerical simulations of optimal equilibrium strategies and the optimal benefit between opinion leaders and Internet users, the optimal trajectory and the steady-state value of the total volume of real information, and the optimal benefit of the false information clarification system are carried out. It is found that under centralized decision-making, equilibrium strategy and total benefit of opinion leaders and Internet users, system total benefit, and total volume of real information can achieve Pareto optimality. Although subsidized decision-making fails to achieve Pareto optimality, with opinion leaders providing cost subsidies for Internet users, it is possible to reach relative Pareto improvement compared with decentralized decision-making.

## 1. Introduction

With the rapid development of the Internet, the public is now able to quickly learn of, and exchange information about, major emergencies in some places via social platforms of new media, such as Facebook, Instagram, Tiktok, and Weibo. With major emergencies featuring abruptness, the wildness of dissemination, and high levels of danger [1], relevant departments find it hard to carry out overall research effectively, therefore failing to release any relevant information to the public in the first place. This leads to a lack of accurate and real information about major emergencies from the public view, who are interested in those emergencies. It is possible that the public, influenced by false information disseminated on social platforms, becomes panicked causing severe online cluster behavior or even the appearance of cluster events, which will obstruct the process of relevant government departments dealing with major emergencies and negatively impact social stability and prosperity. Therefore, to develop countermeasures against false information of major emergencies on social platforms, opinion leaders are encouraged to clarify false information by releasing real information, channeling Internet users' attention, and advising them to adopt real information. It is also very important to establish a false-information clarification system, so that any panic among Internet users could be eliminated, and any repeating impact could be reduced.

Academic circles have carried out multi-perspective research on different clusters' behavior under the background of major emergencies. Kang et al. [2] studied the influence of support provided by e-commerce platforms to businesses after major emergencies. Wu et al. [3] studied social influence and public voice after major emergencies and constructed a public opinion evolution model between the public and the government by means of information entropy. Cao et al. [4] studied the influence of extremist behavior on cluster decision-making and particularly focused on cluster decision-making under major emergencies. Din et al. [5] investigated the impact of Internet customers' behavior on food supply chains. Other scholars carried out research on major emergencies' influence on the public; for example, Akatu et al. [6] studied the public's negative emotions after the COVID-19 pandemic. From the perspective of emergencies, Hong et al. [7] proposed that panic emotions could be disseminated via both the virtual world and the real world when information is exchanged. Liu [8] suggested that the quality of information released by social media is in inverse proportion to the amount of panic behavior caused by major emergencies. McElroy et al. [9] discovered that the level of anxiety caused by major emergencies is related to an individual's age, gender, and health status.

In terms of false-information clarification, Liao and Wang [10] believed that a timely and effective clarification of false information could reduce the negative impact of major emergencies and promote cooperation among the public to disseminate real information. Hosseini and Zandvakili [11] held the view that the dissemination of false information negatively affects the stability of society. Pal and [12] studied individuals with different levels of risk preference and their selection of false information or real information. It was concluded that the individuals were more interested in false information regardless of the level of their risk preference. Bordia et al. [13] proposed effectively limiting the dissemination of false information by clarifying it. Buchanan and Benson [14] found that the dissemination of false information was affected by the level of recognition the receiver had of the disseminator, as well as the receiver's own risk preference. Guess et al. [15] believed that the dissemination of false information affects the public judgment. Vosoughi et al. [16] found that the scope and speed of the dissemination of false information are higher than those of real information. Agarwal et al. [17] thought that after major emergencies, timely clarification of false information should be carried out so that its negative impact could be ameliorated. Ozturk et al. [18] found that clarification of false information could effectively control its dissemination.

Differential game theory has been widely applied in various fields including military science [19], cybernetics [20], science of management [21], and economics [22]. This study mainly applies differential game theory to research on false information clarification systems after major emergencies. Combining classical game theory and cybernetics theory, differential game theory was first applied in modern war to deal with optimal control theory between two or multiple parties. Later, with the improvement of its theoretical framework, differential game theory has become a tool for analyzing different participants' decision behavior. Differential game theory was applied by Shchelchkov [23] to study the issue of chase-and-run among individuals, Machowska et al. [24] to study the business reputation of advertisers, Biancardi et al. [25] to study the recovery of underground water by peasants and water supply institutions, and Garcia-Meza [26] to study the behavior of enterprises and workers in the labor market. To summarize, differential game theory can be regarded as an optimal control process of the gaming interaction among relevant stakeholders. Therefore, this study regards opinion leaders and Internet users involved in social platforms as a false-information clarification system where false-information clarification issues after major emergencies could be studied based on differential game theory. We believe this work is innovative.

This study is based on optimal control theory and differential game theory, and it sets the post-major-emergency impact of multiple false information on the public involved in social platforms as its research object. First of all, three differential game models are constructed under decentralized decision-making, centralized decision-making, and subsidized decision-making between opinion leaders and Internet users; Second, the solution of three differential game models is obtained, and the optimal equilibrium strategies and the respective optimal benefit of opinion leaders and Internet users, the trajectory of the total volume of real information, and the steady-state value and the optimal benefit of the false-information clarification system are analyzed. Finally, a comparative analysis is performed on the three results, and MATLAB numerical simulation software is applied to all key parameters so that the validity of the equilibrium results could be determined. This study provides a vital theoretical basis and decision-making references.

## 2. Problem Description and Basic Assumption

### 2.1. Problem Description

The research objective is a false-information clarification system consisting of a single opinion leader (L) and a single Internet user (U), and the research is carried out under the differential game perspective. After a major emergency begins, many people pay attention to its relative progress, and due to information asymmetry, social platforms are flooded with false information. Therefore, when false information appears, taking self-interest into consideration, opinion leaders could attract Internet users' attention through investigation and evidence collection, so as to obtain more traffic and exposure. Internet users, on the other hand, could have a better sense of participation and the truth of the event through forwarding real information more frequently, so more people would be exposed to it. Benefits are therefore obtained by a sound information dissemination channel created by posting real information.

### 2.2. Model Assumption

**Assumption 1 .**

This study assumes that the effort cost for opinion leaders to release real information is a convex function of the level of their own effort, with diminishing marginal utility; the cost of effort for Internet users to forward real information is a convex function of the level of their own effort, with diminishing marginal utility. The effort cost for opinion leaders and Internet users at time *t* is denoted as *C*_*L*_(*t*) and *C*_*U*_(*t*), and the equations are as follows:

(1) $$\begin{array}{c} \begin{array}{c} C_{L}\left(t\right)=\frac{1}{2}\mu_{L}E_{L}^{2}\left(t\right),\\ C_{U}\left(t\right)=\frac{1}{2}\mu_{U}E_{U}^{2}\left(t\right), \end{array} \end{array}$$

where *μ*_*L*_ and *μ*_*U*_ denote the effort cost coefficients of opinion leaders and Internet users, respectively, with both coefficients being greater than zero; and *E*_*L*_(*t*) and *E*_*U*_(*t*) denote the level of effort of opinion leaders and Internet users at time *t*, respectively.

**Assumption 2 .**

The total volume of real information released on social platforms changes dynamically with time and effort of opinion leaders and Internet users. It is noted that in the real world, some real information fails to reach its expected effect of clarifying false information due to a lack of dissemination. Therefore, it is presumed that the total volume of real information, *R*(*t*), changes with time based on the following dynamic equation:

(2) $$\begin{array}{c} \left\{\begin{array}{c} \dot{R}\left(t\right)=\alpha_{L}E_{L}\left(t\right)+\alpha_{U}E_{U}\left(t\right)-\delta R\left(t\right),\\ R\left(0\right)=R_{0}\geq 0, \end{array}\right. \end{array}$$

where *α*_*L*_ and *α*_*U*_ denote the level of influence of effort by opinion leaders and Internet users on the volume of real information; and *δ* denotes real information's natural dissipation coefficient, with *α*, *β*, and *δ* being greater than zero.

**Assumption 3 .**

Opinion leaders and Internet users could benefit from traffic brought by real information dissemination, the amount of which is co-affected by the initial popularity level of social platforms and the efforts of opinion leaders and Internet users. A similar liner equation for the function of Internet traffic is as follows:

(3) $$\begin{array}{c} F\left(t\right)=f+\gamma \left[\lambda R\left(t\right)+\beta_{L}E_{L}\left(t\right)+\beta_{U}E_{U}\left(t\right)\right]\text{,} \end{array}$$

with *f* denoting the initial popularity level of social platforms, *γ* denoting the level of attention received by major emergencies, *λ* denoting the total volume of real information's influence coefficient on social platforms' traffic, *β*_*L*_ denoting the effort of opinion leaders' influence coefficient on social platforms' traffic, and *β*_*U*_ denoting the effort of Internet users' influence coefficient on social platforms' traffic, with 0 < *γ* ≤ 1 and *β*_*L*_ and *β*_*U*_ being greater than zero. Opinion leaders not only benefit from visitor traffic but also from Internet users directly.

**Assumption 4 .**

The discount rate, *p*, of opinion leaders and Internet users is the same and greater than zero. Behavior strategy selection of both opinion leaders and Internet users is on the basis of maximizing their own interest, with no time limit.

Based on Assumptions 1–4, the objective functions of opinion leaders and Internet users are as follows:

(4) $$\begin{array}{c} \begin{array}{c} J_{L}=\overset{\infty }{\underset{0}{\int }}e^{-\rho t}\left[\omega E_{U}\left(t\right)+\pi_{L}F\left(t\right)-C_{L}\left(t\right)\right]dt,\\ J_{U}=\overset{\infty }{\underset{0}{\int }}e^{-\rho t}\left[\pi_{U}F\left(t\right)-C_{U}\left(t\right)\right]dt, \end{array} \end{array}$$

where *ωE*_*U*_(*t*) denotes benefits of opinion leaders obtained directly from Internet users, *ω* denotes direct benefit per unit gained by opinion leaders from the effort of Internet users, *π*_*L*_*F*(*t*) denotes benefits of opinion leaders obtained from the traffic of visitors on social platforms, *π*_*L*_ denotes benefits of marginal traffic of visitors obtained by opinion leaders, *π*_*U*_*F*(*t*) denotes benefits of Internet users obtained from traffic of visitors on social platforms, and *π*_*U*_ denotes marginal benefits of traffic obtained by Internet users.

All parameters of the models in this study are denoted by constants that do not vary with time, and the game behavior happens in any time period with no limitation. To simplify the writing process, time *t* will not be listed in the following paragraphs.

## 3. Model Construction and Solution

### 3.1. Decentralized Decision-Making of Opinion Leaders and Internet Users

Under decentralized decision-making, opinion leaders and Internet users need to make an independent decision based on the principle of maximizing their own benefits, and the post-game equilibrium strategy is called the Nash equilibrium strategy. Denoting N as a decentralized strategy, at this moment, the decision behavior of opinion leaders and Internet users is as follows:

(5) $$\begin{array}{c} \begin{array}{c} \max_{E_{L}}J_{L}^{N}=\overset{\infty }{\underset{0}{\int }}e^{-\rho t}\left\{\omega E_{U}+\pi_{L}f+\pi_{L}\gamma \left[\lambda R+\beta_{L}E_{L}+\beta_{U}E_{U}\right]-\frac{1}{2}\mu_{L}E_{L}^{2}\right\}dt,\\ \max_{E_{U}}J_{U}^{N}=\overset{\infty }{\underset{0}{\int }}e^{-\rho t}\left\{\pi_{U}f+\pi_{U}\gamma \left[\lambda R+\beta_{L}E_{L}+\beta_{U}E_{U}\right]-\frac{1}{2}\mu_{U}E_{U}^{2}\right\}dt. \end{array} \end{array}$$

**Theorem 1 .**

Under the decentralized decision-making of opinion leaders and Internet users, the equilibrium results are as follows:

(1) The optimal equilibrium strategy for opinion leaders is:
  (6) $$\begin{array}{c} E_{L}^{N^{*}}=\frac{\gamma \pi_{L}\left[\lambda \alpha_{L}+\left(\delta +\rho \right)\beta_{L}\right]}{\left(\delta +\rho \right)\mu_{L}}\text{.} \end{array}$$
(2) The optimal equilibrium strategy for Internet users is:
  (7) $$\begin{array}{c} E_{U}^{N^{*}}=\frac{\gamma \pi_{U}\left[\lambda \alpha_{U}+\left(\delta +\rho \right)\beta_{U}\right]}{\left(\delta +\rho \right)\mu_{U}}\text{.} \end{array}$$
(3) The optimal trajectory of the total volume of real information is:
  (8) $$\begin{array}{c} \left\{\begin{array}{c} R^{N^{*}}=\left(R_{0}-R_{S}^{N}\right)e^{-\delta t}+R_{S}^{N},\\ R_{S}^{N}=\frac{\gamma \alpha_{L}\pi_{L}\left[\lambda \alpha_{L}+\left(\delta +\rho \right)\beta_{L}\right]}{\delta \left(\delta +\rho \right)\mu_{L}}+\frac{\gamma \alpha_{U}\pi_{U}\left[\lambda \alpha_{U}+\left(\delta +\rho \right)\beta_{U}\right]}{\delta \left(\delta +\rho \right)\mu_{U}}, \end{array}\right. \end{array}$$
with *R*_*S*_^*n*^ being the steady-state value of real information under decentralized decision-making.
(4) The optimal benefit for opinion leaders is:
  (9) $$\begin{array}{c} \begin{array}{c} V_{L}^{N^{*}}\left(R\right)=\frac{\lambda \pi_{L}\gamma }{\delta +\rho }R_{S}^{N}+\frac{\pi_{L}f}{\rho }+\frac{\pi_{L}\gamma^{2}\pi_{U}{\left[\lambda \alpha_{U}+\left(\delta +\rho \right)\beta_{U}\right]}^{2}}{\rho {\left(\delta +\rho \right)}^{2}\mu_{U}}\\ +\frac{\gamma^{2}\pi_{L}^{2}{\left[\lambda \alpha_{L}+\left(\delta +\rho \right)\beta_{L}\right]}^{2}}{2\rho {\left(\delta +\rho \right)}^{2}{µ}_{L}}+\frac{\gamma \pi_{U}\omega \left[\lambda \alpha_{U}+\left(\delta +\rho \right)\beta_{U}\right]}{\rho \left(\delta +\rho \right)\mu_{U}}\text{.} \end{array} \end{array}$$
(5) The optimal benefit for Internet users is:
  (10) $$\begin{array}{c} \begin{array}{c} V_{U}^{N^{*}}\left(R\right)=\frac{\lambda \pi_{U}\gamma }{\delta +\rho }R_{S}^{N}+\frac{\pi_{U}f}{\rho }+\frac{\pi_{U}^{2}\gamma^{2}{\left(\lambda \alpha_{U}+\left(\delta +\rho \right)\beta_{U}\right)}^{2}}{2\rho {\left(\delta +\rho \right)}^{2}\mu_{U}}\\ +\frac{\pi_{U}\gamma^{2}\pi_{L}{\left(\lambda \alpha_{L}+\left(\delta +\rho \right)\beta_{L}\right)}^{2}}{\rho {\left(\delta +\rho \right)}^{2}{µ}_{L}} \end{array}\text{.} \end{array}$$
(6) The optimal benefit for the system is:
  (11) $$\begin{array}{c} \left\{\begin{array}{c} V^{N^{*}}\left(R\right)=V_{L}^{N^{*}}\left(R\right)+V_{U}^{N^{*}}\left(R\right)\\ V^{N^{*}}\left(R\right)=\frac{\lambda \gamma \left(\pi_{L}+\pi_{U}\right)}{\delta +\rho }R_{S}^{N}+\frac{f\left(\pi_{L}+\pi_{U}\right)}{\rho }+\frac{\gamma^{2}\pi_{U}\left(\pi_{U}+2\pi_{L}\right){\left[\lambda \alpha_{U}+\left(\delta +\rho \right)\beta_{U}\right]}^{2}}{2\rho {\left(\delta +\rho \right)}^{2}\mu_{U}}\\ +\frac{\gamma^{2}\pi_{L}\left(\pi_{L}+2\pi_{U}\right){\left[\lambda \alpha_{L}+\left(\delta +\rho \right)\beta_{L}\right]}^{2}}{2\rho {\left(\delta +\rho \right)}^{2}{µ}_{L}}+\frac{\gamma \pi_{U}\omega \left[\lambda \alpha_{U}+\left(\delta +\rho \right)\beta_{U}\right]}{\rho \left(\delta +\rho \right)\mu_{U}}\text{.} \end{array}\right. \end{array}$$

**Verification 1.**

According to optimal control theory, if *R* ≥ 0, then both *V*_*L*_^*N*^(*R*) and *V*_*U*_^*N*^(*R*) satisfy the Hamilton–Jacobi–Bellman (HJB) equation, and both have first and second derivatives, namely:

(12) $$\begin{array}{c} \rho V_{L}^{N}\left(R\right)=\max_{E_{L}}\left\{\begin{array}{c} \omega E_{U}+\pi_{L}f+\pi_{L}\gamma \left[\lambda R+\beta_{L}E_{L}+\beta_{U}E_{U}\right]\\ -\frac{1}{2}\mu_{L}E_{L}^{2}+V_{L}^{N^{\prime }}\left[\alpha_{L}E_{L}+\alpha_{U}E_{U}-\delta R\right] \end{array}\right\}\text{,} \end{array}$$

(13) $$\begin{array}{c} \rho V_{U}^{N}\left(R\right)=\max_{E_{U}}\left\{\begin{array}{c} \pi_{U}f+\pi_{U}\gamma \left[\lambda R+\beta_{L}E_{L}+\beta_{U}E_{U}\right]\\ -\frac{1}{2}\mu_{U}E_{U}^{2}+V_{U}^{N^{\prime }}\left[\alpha_{L}E_{L}+\alpha_{U}E_{U}-\delta R\right] \end{array}\right\}\text{.} \end{array}$$

To solve *E*_L_, the first derivative of the function on the right side of equation (12) is taken and set equal to zero, which gives:

(14) $$\begin{array}{c} E_{L}^{N}=\frac{\pi_{L}\gamma \beta_{L}+\alpha_{L}V_{L}^{N^{\prime }}}{\mu_{L}}\text{.} \end{array}$$

To solve *E*_U_, the first derivative of the function on the right of the equation (13) is taken and set equal to zero, which gives:

(15) $$\begin{array}{c} E_{U}^{N}=\frac{\pi_{U}\gamma \beta_{U}+\alpha_{U}V_{U}^{N^{\prime }}}{\mu_{U}}\text{.} \end{array}$$

After inputting equations (14) and (15) in to (12), it is concluded that:

(16) $$\begin{array}{c} \begin{array}{c} \rho V_{L}^{N}\left(R\right)=\left(\lambda \pi_{L}\gamma -\delta V_{L}^{N^{\prime }}\right)R+V_{L}^{N^{\prime }}\left[\frac{\alpha_{L}\left(\alpha_{L}V_{L}^{N^{\prime }}+\beta_{L}\pi_{L}\gamma \right)}{\mu_{L}}+\frac{\alpha_{U}\left(\alpha_{U}V_{U}^{N^{\prime }}+\beta_{U}\pi_{U}\gamma \right)}{\mu_{U}}\right]\\ +\pi_{L}f-\frac{{\left(\alpha_{L}V_{L}^{N^{\prime }}+\beta_{L}\pi_{L}\gamma \right)}^{2}}{2\mu_{L}}+\frac{\omega \left(\alpha_{U}V_{U}^{N^{\prime }}+\beta_{U}\pi_{U}\gamma \right)}{\mu_{U}}\\ +\pi_{L}\gamma \left[\frac{\beta_{L}\left(\alpha_{L}V_{L}^{N^{\prime }}+\beta_{L}\pi_{L}\gamma \right)}{\mu_{L}}+\frac{\beta_{U}\left(\alpha_{U}V_{U}^{N^{\prime }}+\beta_{U}\pi_{U}\gamma \right)}{\mu_{U}}\right]. \end{array} \end{array}$$

Inputting equations (14) and (15) in to (13), it is concluded that:

(17) $$\begin{array}{c} \begin{array}{c} \rho V_{U}^{N}\left(R\right)=\left(\lambda \pi_{U}\gamma -\delta V_{U}^{N^{\prime }}\right)R+V_{U}^{N^{\prime }}\left[\frac{\alpha_{L}\left(\alpha_{L}V_{L}^{N^{\prime }}+\beta_{L}\pi_{L}\gamma \right)}{\mu_{L}}+\frac{\alpha_{U}\left(\alpha_{U}V_{U}^{N^{\prime }}+\beta_{U}\pi_{U}\gamma \right)}{\mu_{U}}\right]\\ +\pi_{U}f-\frac{{\left(\alpha_{U}V_{U}^{N^{\prime }}+\beta_{U}\pi_{U}\gamma \right)}^{2}}{2\mu_{U}}\\ +\pi_{U}\gamma \left[\frac{\beta_{L}\left(\alpha_{L}V_{L}^{N^{\prime }}+\beta_{L}\pi_{L}\gamma \right)}{\mu_{L}}+\frac{\beta_{U}\left(\alpha_{U}V_{U}^{N^{\prime }}+\beta_{U}\pi_{U}\gamma \right)}{\mu_{U}}\right]\text{.} \end{array} \end{array}$$

From the structural characteristics of equations (16) and (17), the linear optimal value function for *R* is the solution of the HJB equation. Therefore, it is assumed that the analytic equations of *V*_*L*_^*N*^(*R*) and *V*_*U*_^*N*^(*R*) for *R*are:

(18) $$\begin{array}{c} V_{L}^{N}\left(R\right)=a_{1}R+a_{2}\text{,} \end{array}$$

(19) $$\begin{array}{c} V_{U}^{N}\left(R\right)=b_{1}R+b_{2}\text{,} \end{array}$$

with *a*_1_, *a*_2_, *b*_1_, and *b*_2_ being undetermined coefficients. It is further concluded from equations (18) and (19) that:

(20) $$\begin{array}{c} V_{L}^{N^{\prime }}\left(R\right)=a_{1}\text{,} \end{array}$$

(21) $$\begin{array}{c} V_{U}^{N^{\prime }}\left(R\right)=b_{1}\text{.} \end{array}$$

Equations (18)–(21) are inputted into equations (16) and (17). Using the method of undetermined coefficients, the values of *a*_1_, *a*_2_, *b*_1_, and *b*_2_ could be obtained:

(22) $$\begin{array}{c} a_{1}=\frac{\lambda \pi_{L}\gamma }{\delta +\rho }\text{,} \end{array}$$

(23) $$\begin{array}{c} \begin{array}{c} a_{2}=\frac{\pi_{L}f}{\rho }+\frac{\pi_{L}\gamma^{2}\pi_{U}{\left[\lambda \alpha_{U}+\left(\delta +\rho \right)\beta_{U}\right]}^{2}}{\rho {\left(\delta +\rho \right)}^{2}\mu_{U}}\\ +\frac{\gamma^{2}\pi_{L}^{2}{\left[\lambda \alpha_{L}+\left(\delta +\rho \right)\beta_{L}\right]}^{2}}{2\rho {\left(\delta +\rho \right)}^{2}{µ}_{L}}+\frac{\gamma \pi_{U}\omega \left[\lambda \alpha_{U}+\left(\delta +\rho \right)\beta_{U}\right]}{\rho \left(\delta +\rho \right)\mu_{U}}\text{,} \end{array} \end{array}$$

(24) $$\begin{array}{c} b_{1}=\frac{\lambda \pi_{U}\gamma }{\delta +\rho }\text{,} \end{array}$$

(25) $$\begin{array}{c} b_{2}=\frac{\pi_{U}f}{\rho }+\frac{\pi_{U}^{2}\gamma^{2}{\left[\lambda \alpha_{U}+\left(\delta +\rho \right)\beta_{U}\right]}^{2}}{2\rho {\left(\delta +\rho \right)}^{2}\mu_{U}}+\frac{\pi_{U}\gamma^{2}\pi_{L}{\left[\lambda \alpha_{L}+\left(\delta +\rho \right)\beta_{L}\right]}^{2}}{\rho {\left(\delta +\rho \right)}^{2}{µ}_{L}}\text{.} \end{array}$$

Inputting equations (20) and (22) into equation (14), the optimal equilibrium strategy for opinion leaders is shown as equation (6); inputting equations (21) and (24) into equation (15), the optimal equilibrium strategy for Internet users is shown as equation (7); inputting equations (6) and (7) into equation (2), the optimal trajectory of the total volume of real information and the steady-state value are shown as equation (8); inputting equations (22) and (23) into equation (18), the optimal benefit for opinion leaders is shown as equation (9); inputting equations (24) and (25) into equation (19), the optimal benefit for Internet users is shown as equation (10); from equations (9) and (10), the optimal benefit for the whole false information clarification system is shown as equation (11). So far, theorem 1 has been verified.

**Deduction 1 .**

From Theorem 1, it is known that under the context of the decentralized decision, both Internet users and opinion leaders make their decision based on the maximum of their own benefit, and the decision behavior of both sides has no impact on the other. The optimal equilibrium of both sides (i.e., the level of their effort) is simultaneously affected by: the level of attention gained after major emergencies, each side's marginal traffic benefit, influential coefficients of the total volume of real information, and effort level of each side on social platform traffic, and effort level of each side's influential coefficient on the total volume of real information and each side's effort cost coefficient.

The total volume of real information is decided by the effort level of both opinion leaders and Internet users. The optimal benefit of opinion leaders, Internet users, and false information clarification systems increases with the growth of the total volume of real information. This means that the more effort opinion leaders and Internet users pay, the more benefit they will get. A detailed interrelated relationship is shown in Table 1.

### 3.2. Centralized Decision-Making of Opinion Leaders and Internet Users

Under the context of centralized decision-making, meaning cooperation, opinion leaders and Internet users, as a cooperative system who shares the same goal, aim to maximize system benefit while making decisions. The post-game equilibrium strategy is called the Nash equilibrium strategy. Denoting *C* as centralized decision-making, the decision behavior of the current system is:

(26) $$\begin{array}{c} \max_{E_{L},E_{U}}J_{S}^{C}=\overset{\infty }{\underset{0}{\int }}e^{-\rho t}\left\{\begin{array}{c} \omega E_{U}-\frac{1}{2}\mu_{L}E_{L}^{2}-\frac{1}{2}\mu_{U}E_{U}^{2}\\ +\left(\pi_{L}+\pi_{U}\right)\left[f+\gamma \left(\lambda R+\beta_{L}E_{L}+\beta_{U}E_{U}\right)\right] \end{array}\right\}dt\text{.} \end{array}$$

**Theorem 2 .**

The equilibrium result under centralized decision-making of opinion leaders and Internet users is:

(1) The optimal equilibrium strategy of opinion leaders is:
  (27) $$\begin{array}{c} E_{L}^{C^{*}}=\frac{\gamma \left(\pi_{L}+\pi_{U}\right)\left[\lambda \alpha_{L}+\left(\delta +\rho \right)\beta_{L}\right]}{\mu_{L}\left(\delta +\rho \right)}\text{.} \end{array}$$
(2) The optimal equilibrium strategy of Internet users is:
  (28) $$\begin{array}{c} E_{U}^{C^{*}}=\frac{\omega \left(\delta +\rho \right)+\gamma \left(\pi_{L}+\pi_{U}\right)\left[\lambda \alpha_{U}+\left(\delta +\rho \right)\beta_{U}\right]}{\mu_{U}\left(\delta +\rho \right)}\text{.} \end{array}$$
(3) The optimal trajectory of the total volume of real information is:
  (29) $$\begin{array}{c} \left\{\begin{array}{c} R^{C^{*}}=\left(R_{0}-R_{S}^{C}\right)e^{-\delta t}+R_{S}^{C}\\ \begin{array}{c} R_{S}^{C}=\frac{\gamma \alpha_{L}\left(\pi_{L}+\pi_{U}\right)\left[\lambda \alpha_{L}+\left(\delta +\rho \right)\beta_{L}\right]}{\delta \mu_{L}\left(\delta +\rho \right)}\\ +\frac{\omega \alpha_{U}\left(\delta +\rho \right)+\gamma \alpha_{U}\left(\pi_{L}+\pi_{U}\right)\left[\lambda \alpha_{U}+\left(\delta +\rho \right)\beta_{U}\right]}{\delta \mu_{U}\left(\delta +\rho \right)}\text{.} \end{array} \end{array}\right. \end{array}$$
with *R*_*S*_^*C*^ being the steady-state value of real information under centralized decision-making.
(4) The optimal benefit of the system is:
  (30) $$\begin{array}{c} \begin{array}{c} V^{C^{*}}\left(R\right)=\frac{\lambda \gamma \left(\pi_{L}+\pi_{U}\right)}{\delta +\rho }R_{S}^{C}+\frac{\omega \gamma \left(\pi_{L}+\pi_{U}\right)\left[\lambda \alpha_{U}+\left(\delta +\rho \right)\beta_{U}\right]}{\rho \left(\delta +\rho \right)\mu_{U}}\\ +\frac{f\left(\pi_{L}+\pi_{U}\right)}{\rho }+\frac{\gamma^{2}{\left(\pi_{L}+\pi_{U}\right)}^{2}{\left[\lambda \alpha_{U}+\left(\delta +\rho \right)\beta_{U}\right]}^{2}}{2\rho {\left(\delta +\rho \right)}^{2}\mu_{U}}\\ +\frac{\gamma^{2}{\left(\pi_{L}+\pi_{U}\right)}^{2}{\left[\lambda \alpha_{L}+\left(\delta +\rho \right)\beta_{L}\right]}^{2}}{2\rho {\left(\delta +\rho \right)}^{2}{µ}_{L}}+\frac{\omega^{2}}{2\rho \mu_{U}} \end{array}\text{.} \end{array}$$

**Verification 2.**

According to optimal control theory, if *R* ≥ 0, then *V*_*S*_^*C*^(*R*) satisfies the HJB equation, and *V*_*S*_^*C*^(*R*) has first and second derivatives, namely:

(31) $$\begin{array}{c} \rho V_{S}^{C}\left(R\right)=\max_{E_{L},E_{U}}\left\{\begin{array}{c} \omega E_{U}-\frac{1}{2}\mu_{L}E_{L}^{2}-\frac{1}{2}\mu_{U}E_{U}^{2}+\left(\pi_{L}+\pi_{U}\right)\left[f+\gamma \left(\lambda R+\beta_{L}E_{L}+\beta_{U}E_{U}\right)\right]\\ +V_{S}^{C^{\prime }}\left[\alpha_{L}E_{L}+\alpha_{U}E_{U}-\delta R\right] \end{array}\right\}\text{.} \end{array}$$

To solve *E*_L_ and *E*_U_, the first derivatives of the function on the right side of the equations are taken and set equal to zero, which gives:

(32) $$\begin{array}{c} E_{L}^{C}=\frac{\left(\pi_{L}+\pi_{U}\right)\gamma \beta_{L}+\alpha_{L}V_{S}^{C^{\prime }}}{\mu_{L}}\text{,} \end{array}$$

(33) $$\begin{array}{c} E_{U}^{C}=\frac{\omega +\left(\pi_{L}+\pi_{U}\right)\gamma \beta_{U}+\alpha_{U}V_{S}^{C^{\prime }}}{\mu_{U}}\text{.} \end{array}$$

Inputting equations (32) and (33) into equation (31), it is concluded that:

(34) $$\begin{array}{c} \begin{array}{c} \rho V_{S}^{C}\left(R\right)=\left[\lambda \gamma \left(\pi_{L}+\pi_{U}\right)-\delta V_{S}^{C^{\prime }}\right]R+\frac{\omega \left[\omega +\alpha_{U}V_{S}^{C^{\prime }}+\beta_{U}\gamma \left(\pi_{L}+\pi_{U}\right)\right]}{\mu_{U}}\\ +V_{S}^{C^{\prime }}\left\{\frac{\alpha_{L}\left[\alpha_{L}V_{S}^{C^{\prime }}+\beta_{L}\gamma \left(\pi_{L}+\pi_{U}\right)\right]}{\mu_{L}}+\frac{\alpha_{U}\left[\omega +\alpha_{U}V_{S}^{C^{\prime }}+\beta_{U}\gamma \left(\pi_{L}+\pi_{U}\right)\right]}{\mu_{U}}\right\}\\ +\left(\pi_{L}+\pi_{U}\right)\left\{f+\gamma \left[\begin{array}{c} \frac{\beta_{L}\left[\alpha_{L}V_{S}^{C^{\prime }}+\beta_{L}\gamma \left(\pi_{L}+\pi_{U}\right)\right]}{\mu_{L}}\\ +\frac{\beta_{U}\left[\omega +\alpha_{U}V_{S}^{C^{\prime }}+\beta_{U}\gamma \left(\pi_{L}+\pi_{U}\right)\right]}{\mu_{U}} \end{array}\right]\right\}\\ -\frac{{\left[\omega +\alpha_{U}V_{S}^{C^{\prime }}+\beta_{U}\gamma \left(\pi_{L}+\pi_{U}\right)\right]}^{2}}{2\mu_{U}}-\frac{{\left[\alpha_{L}V_{S}^{C^{\prime }}+\beta_{L}\gamma \left(\pi_{L}+\pi_{U}\right)\right]}^{2}}{2\mu_{L}}\text{.} \end{array} \end{array}$$

From the structural characteristics of equation (34), it is assumed that the analytic equation of *V*_*S*_^*C*^(*R*) for *R* is:

(35) $$\begin{array}{c} V_{S}^{C}\left(R\right)=c_{1}R+c_{2}\text{,} \end{array}$$

with *c*_1_ and *c*_2_ being undetermined coefficients. It is further obtained from equation (35) that:

(36) $$\begin{array}{c} V_{S}^{C^{\prime }}\left(R\right)=c_{1}\text{.} \end{array}$$

Equations (35) and (36) are inputted into equation (34). According to the method of undetermined coefficients, the values of *c*_1_ and *c*_2_ could be obtained:

(37) $$\begin{array}{c} c_{1}=\frac{\lambda \gamma \left(\pi_{L}+\pi_{U}\right)}{\delta +\rho }\text{,} \end{array}$$

(38) $$\begin{array}{c} \begin{array}{c} c_{2}=\frac{\gamma^{2}{\left(\pi_{L}+\pi_{U}\right)}^{2}{\left[\lambda \alpha_{L}+\left(\delta +\rho \right)\beta_{L}\right]}^{2}}{2\rho {\left(\delta +\rho \right)}^{2}{µ}_{L}}+\frac{\gamma^{2}{\left(\pi_{L}+\pi_{U}\right)}^{2}{\left[\lambda \alpha_{U}+\left(\delta +\rho \right)\beta_{U}\right]}^{2}}{2\rho {\left(\delta +\rho \right)}^{2}\mu_{U}}\\ +\frac{f\left(\pi_{L}+\pi_{U}\right)}{\rho }+\frac{\omega^{2}}{2\rho \mu_{U}}+\frac{\omega \gamma \left(\pi_{L}+\pi_{U}\right)\left[\lambda \alpha_{U}+\left(\delta +\rho \right)\beta_{U}\right]}{\rho \left(\delta +\rho \right)\mu_{U}}\text{.} \end{array} \end{array}$$

Inputting equations (36) and (37) into equation (32), the optimal equilibrium strategy for opinion leaders is shown as equation (27); inputting equations (36) and (37) into equation (33), the optimal equilibrium strategy for Internet users is shown as equation (28); inputting equations (27) and (28) into equation (2), the optimal trajectory of total volume of real information and the steady-state value are shown as equation (29); inputting equations (37) and (38) into equation (35), the optimal benefit for the whole false information clarification system is shown as equation (30). So far, theorem 2 has been verified.

**Deduction 2 .**

From Theorem 2, it is known that under the context of the centralized decision, both Internet users and opinion leaders make their decision based on the maximum of false information clarification system's benefit, and the decision behavior of both sides is interrelated. Compared with decentralized decision, the optimal equilibrium strategy of opinion leaders (i.e., effort level of themselves) is additionally affected by the marginal traffic benefit of Internet users; the optimal equilibrium strategy of Internet users (i.e., effort level of themselves) is additionally affected by opinion leaders' marginal traffic benefit and direct benefit per unit gained from Internet users' effort. The optimal trajectory of the total volume of real information is dependent on the effort paid by opinion leaders and Internet users, while the optimal benefit of the false information clarification system increases with the growth of the total volume of real information. This means that the more effort paid by opinion leaders and Internet users, the more benefit they will receive. A detailed interrelated relationship is shown in Table 2.

### 3.3. Cost-Subsidized Decision-Making of Opinion Leaders and Internet Users

In the real world, opinion leaders could attract Internet users' attention using the method of “Forwarding + Commenting, drawing a lottery.” With rewards from opinion leaders, Internet users are more willing to forward and spread real information. This study refers to such reward behaviors of opinion leaders as a cost subsidy.

Under such a decision-making behavior, a Stackelberg subordinate game model is established, with opinion leaders being leaders and Internet users being followers. The whole decision-making process is divided into two phases: in the first phrase, opinion leaders confirm their own effort level as *E*_*L*_^*S*^ and provide a cost subsidy for Internet users who forward and spread information. The proportion of the cost subsidy provided by opinion leaders to Internet users is *ε*(0 ≤ *ε* ≤ 1); in the second phrase, based on the obtained cost subsidy and decision-making behavior of opinion leaders, Internet users confirm their own effort level as *E*_*U*_^*S*^. The post-game equilibrium strategy is called the Stackelberg equilibrium strategy. Denoting S as opinion leaders' cost-subsidy decision, the decision-making behavior of both opinion leaders and Internet users at this moment is:

(39) $$\begin{array}{c} \begin{array}{c} \max_{E_{L},\varepsilon }J_{L}^{S}=\overset{\infty }{\underset{0}{\int }}e^{-\rho t}\left\{\omega E_{U}+\pi_{L}f+\pi_{L}\gamma \left[\lambda R+\beta_{L}E_{L}+\beta_{U}E_{U}\right]-\frac{1}{2}\mu_{L}E_{L}^{2}-\frac{1}{2}\varepsilon \mu_{U}E_{U}^{2}\right\}dt,\\ \max_{E_{U}}J_{U}^{S}=\overset{\infty }{\underset{0}{\int }}e^{-\rho t}\left\{\pi_{U}f+\pi_{U}\gamma \left[\lambda R+\beta_{L}E_{L}+\beta_{U}E_{U}\right]-\frac{1}{2}\left(1-\varepsilon \right)\mu_{U}E_{U}^{2}\right\}dt\text{.} \end{array} \end{array}$$

**Theorem 3 .**

The equilibrium results under the cost-subsidy decision of opinion leaders and Internet users are:

(1) The cost-subsidy proportion of opinion leaders is:
  (40) $$\begin{array}{c} \begin{array}{c} \varepsilon^{*}=\left\{\begin{array}{c} \frac{2\left(\delta +\rho \right)\omega +\gamma \left(2\pi_{L}-\pi_{U}\right)\left[\lambda \alpha_{U}+\left(\delta +\rho \right)\beta_{U}\right]}{2\left(\delta +\rho \right)\omega +\gamma \left(2\pi_{L}+\pi_{U}\right)\left[\lambda \alpha_{U}+\left(\delta +\rho \right)\beta_{U}\right]},D<1,\\ 0,D\geq 1\text{.} \end{array}\right.\\ D=\frac{\gamma \left(\pi_{U}-2\pi_{L}\right)\left[\lambda \alpha_{U}+\left(\delta +\rho \right)\beta_{U}\right]}{2\left(\delta +\rho \right)\omega }. \end{array} \end{array}$$
(2) The optimal equilibrium strategy of opinion leaders is:
  (41) $$\begin{array}{c} E_{L}^{S^{*}}=\frac{\gamma \pi_{L}\left[\lambda \alpha_{L}+\left(\delta +\rho \right)\beta_{L}\right]}{\left(\delta +\rho \right)\mu_{L}}\text{.} \end{array}$$
(3) The optimal equilibrium strategy of Internet users is:
  (42) $$\begin{array}{c} \begin{array}{c} E_{U}^{S^{*}}=\frac{\gamma \pi_{U}\left[\lambda \alpha_{U}+\left(\delta +\rho \right)\beta_{U}\right]}{\left(1-\varepsilon \right)\left(\delta +\rho \right)\mu_{U}}\\ =\frac{2\left(\delta +\rho \right)\omega +\gamma \left(2\pi_{L}+\pi_{U}\right)\left[\lambda \alpha_{U}+\left(\delta +\rho \right)\beta_{U}\right]}{2\left(\delta +\rho \right)\mu_{U}}\text{.} \end{array} \end{array}$$
(4) The optimal trajectory of the total volume of real information is:
  (43) $$\begin{array}{c} \begin{array}{c} R^{S^{*}}=\left(R_{0}-R_{S}^{S}\right)e^{-\delta t}+R_{S}^{S}\\ R_{S}^{S}=\frac{\gamma \alpha_{L}\pi_{L}\left[\lambda \alpha_{L}+\left(\delta +\rho \right)\beta_{L}\right]}{\delta \left(\delta +\rho \right)\mu_{L}}+\frac{\gamma \alpha_{U}\pi_{U}\left[\lambda \alpha_{U}+\left(\delta +\rho \right)\beta_{U}\right]}{\delta \left(1-\varepsilon \right)\left(\delta +\rho \right)\mu_{U}}\\ =\frac{\gamma \alpha_{L}\pi_{L}\left[\lambda \alpha_{L}+\left(\delta +\rho \right)\beta_{L}\right]}{\delta \left(\delta +\rho \right)\mu_{L}}\\ +\frac{\alpha_{U}\left\{2\left(\delta +\rho \right)\omega +\gamma \left(2\pi_{L}+\pi_{U}\right)\left[\lambda \alpha_{U}+\left(\delta +\rho \right)\beta_{U}\right]\right\}}{2\delta \left(\delta +\rho \right)\mu_{U}}. \end{array} \end{array}$$
with *R*_*S*_^*S*^ being the steady-state value of the total volume of real information under the cost-subsidy decision.
(5) The optimal benefit of opinion leaders is:
  (44) $$\begin{array}{c} \begin{array}{c} V_{L}^{S^{*}}\left(R\right)=\frac{\lambda \pi_{L}\gamma }{\delta +\rho }R_{S}^{S}+\frac{\pi_{L}\gamma^{2}\pi_{U}{\left[\lambda \alpha_{U}+\left(\delta +\rho \right)\beta_{U}\right]}^{2}}{\rho \left(1-\varepsilon \right){\left(\delta +\rho \right)}^{2}\mu_{U}}+\frac{\gamma^{2}\pi_{L}^{2}{\left[\lambda \alpha_{L}+\left(\delta +\rho \right)\beta_{L}\right]}^{2}}{2\rho {\left(\delta +\rho \right)}^{2}{µ}_{L}}\\ +\frac{\omega \gamma \pi_{U}\left[\lambda \alpha_{U}+\left(\delta +\rho \right)\beta_{U}\right]}{\rho \left(1-\varepsilon \right)\left(\delta +\rho \right)\mu_{U}}-\frac{\gamma^{2}\pi_{U}^{2}\varepsilon {\left[\lambda \alpha_{U}+\left(\delta +\rho \right)\beta_{U}\right]}^{2}}{2\rho {\left(1-\varepsilon \right)}^{2}{\left(\delta +\rho \right)}^{2}\mu_{U}}+\frac{\pi_{L}f}{\rho }\\ =\frac{\lambda \pi_{L}\gamma }{\delta +\rho }R_{S}^{S}+\frac{\alpha_{U}^{2}\lambda^{2}\gamma^{2}{\left(2\pi_{L}+\pi_{U}\right)}^{2}}{8\rho {\left(\delta +\rho \right)}^{2}\mu_{U}}+\frac{{\left[\alpha_{L}\lambda +\beta_{L}\left(\delta +\rho \right)\right]}^{2}\pi_{L}^{2}\gamma^{2}}{2\rho {\left(\delta +\rho \right)}^{2}{µ}_{L}}+\frac{\pi_{L}f}{\rho }\\ +\frac{\gamma \beta_{U}\left(2\pi_{L}+\pi_{U}\right)\left[\beta_{U}\left(2\pi_{L}+\pi_{U}\right)\gamma +4\omega \right]+4\omega^{2}}{8\rho \mu_{U}}\\ +\frac{\lambda \gamma \alpha_{U}\left(2\pi_{L}+\pi_{U}\right)\left[2\omega +\gamma \beta_{U}\left(2\pi_{L}+\pi_{U}\right)\right]}{4\rho \left(\delta +\rho \right)\mu_{U}}\text{.} \end{array} \end{array}$$
(6) The optimal benefit of Internet users is:
  (45) $$\begin{array}{c} \begin{array}{c} V_{U}^{S^{*}}\left(R\right)=\frac{\lambda \pi_{U}\gamma }{\delta +\rho }R_{S}^{S}+\frac{\pi_{U}\gamma^{2}\pi_{L}{\left[\lambda \alpha_{L}+\left(\delta +\rho \right)\beta_{L}\right]}^{2}}{\rho {\left(\delta +\rho \right)}^{2}{µ}_{L}}\\ +\frac{\pi_{U}f}{\rho }+\frac{\pi_{U}^{2}\gamma^{2}{\left[\lambda \alpha_{U}+\left(\delta +\rho \right)\beta_{U}\right]}^{2}}{2\rho \left(1-\varepsilon \right){\left(\delta +\rho \right)}^{2}\mu_{U}}\\ =\frac{\lambda \pi_{U}\gamma }{\delta +\rho }R_{S}^{S}+\frac{\pi_{U}\beta_{U}^{2}\left(2\pi_{L}+\pi_{U}\right)\gamma^{2}}{4\rho \mu_{U}}+\frac{{\left[\alpha_{L}\lambda +\beta_{L}\left(\delta +\rho \right)\right]}^{2}\pi_{U}\pi_{L}\gamma^{2}}{\rho {\left(\delta +\rho \right)}^{2}{µ}_{L}}\\ +\frac{\pi_{U}\alpha_{U}^{2}\lambda^{2}\gamma^{2}\left(2\pi_{L}+\pi_{U}\right)}{4\rho {\left(\delta +\rho \right)}^{2}\mu_{U}}+\frac{f\pi_{U}}{\rho }\\ +\frac{\pi_{U}\gamma \left\{\alpha_{U}\lambda \omega +\beta_{U}\left[\alpha_{U}\lambda \left(2\pi_{L}+\pi_{U}\right)\gamma +\left(\delta +\rho \right)\omega \right]\right\}}{2\rho \left(\delta +\rho \right)\mu_{U}}\text{.} \end{array} \end{array}$$
(7) The optimal benefit of the system is:
  (46) $$\begin{array}{c} \begin{array}{c} V^{S^{*}}\left(R\right)=V_{L}^{S^{*}}\left(R\right)+V_{U}^{S^{*}}\left(R\right)\\ =\frac{\lambda \gamma \left(\pi_{L}+\pi_{U}\right)}{\delta +\rho }R_{S}^{S}+\frac{\omega \gamma \pi_{U}\left[\lambda \alpha_{U}+\left(\delta +\rho \right)\beta_{U}\right]}{\rho \left(1-\varepsilon \right)\left(\delta +\rho \right)\mu_{U}}+\frac{f\left(\pi_{L}+\pi_{U}\right)}{\rho }\\ +\frac{\gamma^{2}\pi_{L}\left[2\pi_{U}+\pi_{L}\right]{\left[\lambda \alpha_{L}+\left(\delta +\rho \right)\beta_{L}\right]}^{2}}{2\rho {\left(\delta +\rho \right)}^{2}{µ}_{L}}-\frac{\gamma^{2}\pi_{U}^{2}\varepsilon {\left[\lambda \alpha_{U}+\left(\delta +\rho \right)\beta_{U}\right]}^{2}}{2\rho {\left(1-\varepsilon \right)}^{2}{\left(\delta +\rho \right)}^{2}\mu_{U}}\\ +\frac{\gamma^{2}\pi_{U}\left[2\pi_{L}+\pi_{U}\right]{\left[\lambda \alpha_{U}+\left(\delta +\rho \right)\beta_{U}\right]}^{2}}{2\rho \left(1-\varepsilon \right){\left(\delta +\rho \right)}^{2}\mu_{U}}\\ =\frac{\lambda \gamma \left(\pi_{L}+\pi_{U}\right)}{\delta +\rho }R_{S}^{S}+\frac{f\left(\pi_{L}+\pi_{U}\right)}{\rho }+\frac{\left(2\pi_{L}+3\pi_{U}\right)\left(2\pi_{L}+\pi_{U}\right)\alpha_{U}^{2}\lambda^{2}\gamma^{2}}{8\rho {\left(\delta +\rho \right)}^{2}\mu_{U}}\\ +\frac{4\omega^{2}+\gamma \beta_{U}\left(2\pi_{L}+\pi_{U}\right)\left[\left(2\pi_{L}+3\pi_{U}\right)\gamma \beta_{U}+4\omega \right]}{8\rho \mu_{U}}\\ +\frac{\left(2\pi_{U}+\pi_{L}\right)\pi_{L}\gamma^{2}{\left[\alpha_{L}\lambda +\beta_{L}\left(\delta +\rho \right)\right]}^{2}}{2\rho {\left(\delta +\rho \right)}^{2}{µ}_{L}}\\ +\frac{\omega 2\pi_{U}\gamma \left[\alpha_{U}\lambda +\beta_{U}\left(\delta +\rho \right)\right]+\lambda \gamma \alpha_{U}\left(2\pi_{L}+\pi_{U}\right)\left[2\omega +\left(2\pi_{L}+3\pi_{U}\right)\gamma \beta_{U}\right]}{4\rho \left(\delta +\rho \right)\mu_{U}}\text{.} \end{array} \end{array}$$

**Verification 3.**

To obtain the equilibrium solution of the Stackelberg subordinate game, the optimal control issue of Internet users should be addressed first with the backward induction method. According to optimal control theory, if *R* ≥ 0, then *V*_*U*_^*S*^(*R*) satisfies the HJB equation, and *V*_*U*_^*S*^(*R*) has first and second derivatives, namely:

(47) $$\begin{array}{c} \rho V_{U}^{S}\left(R\right)=\max_{E_{U}}\left\{\begin{array}{c} \pi_{U}f+\pi_{U}\gamma \left[\lambda R+\beta_{L}E_{L}+\beta_{U}E_{U}\right]\\ -\frac{1}{2}\left(1-\varepsilon \right)\mu_{U}E_{U}^{2}+V_{U}^{S^{\prime }}\left[\alpha_{L}E_{L}+\alpha_{U}E_{U}-\delta R\right] \end{array}\right\}\text{.} \end{array}$$

To solve *E*_U_, the first derivative of the function on the right side of the equation is taken and set equal to zero, which gives:

(48) $$\begin{array}{c} E_{U}^{S}=\frac{\pi_{U}\gamma \beta_{U}+\alpha_{U}V_{U}^{S^{\prime }}}{\mu_{U}\left(1-\varepsilon \right)}\text{.} \end{array}$$

To maximize their own interest, opinion leaders would predict the behavior strategy of Internet users so as to confirm their own effort level and cost-subsidy proportion. At this moment, the HJB equation for opinion leaders is:

(49) $$\begin{array}{c} \rho V_{L}^{S}\left(R\right)=\max_{E_{L},\varepsilon }\left\{\begin{array}{c} \omega E_{U}+\pi_{L}f+\pi_{L}\gamma \left[\lambda R+\beta_{L}E_{L}+\beta_{U}E_{U}\right]-\frac{1}{2}\mu_{L}E_{L}^{2}\\ -\frac{1}{2}\varepsilon \mu_{U}E_{U}^{2}+{V_{L}^{S}}^{\prime }\left[\alpha_{L}E_{L}+\alpha_{U}E_{U}-\delta R\right] \end{array}\right\}\text{,} \end{array}$$

To solve *E*_L_, the first derivative of the function on the right side of equation (49) is taken and set equal to zero, which gives:

(50) $$\begin{array}{c} E_{L}^{S}=\frac{\pi_{L}\gamma \beta_{L}+\alpha_{L}V_{L}^{S^{\prime }}}{\mu_{L}}\text{.} \end{array}$$

Inputting equation (48) into (49), it is concluded that:

(51) $$\begin{array}{c} \rho V_{L}^{S}\left(R\right)=\max_{E_{L},\varepsilon }\left\{\begin{array}{c} \frac{\omega \left(\pi_{U}\gamma \beta_{U}+\alpha_{U}V_{U}^{S^{\prime }}\right)}{\mu_{U}\left(1-\varepsilon \right)}+\pi_{L}\gamma \left[\lambda R+\beta_{L}E_{L}+\frac{\beta_{U}\left(\pi_{U}\gamma \beta_{U}+\alpha_{U}V_{U}^{S^{\prime }}\right)}{\mu_{U}\left(1-\varepsilon \right)}\right]\\ -\frac{1}{2}\mu_{L}E_{L}^{2}-\frac{1}{2}\varepsilon \mu_{U}{\left[\frac{\pi_{U}\gamma \beta_{U}+\alpha_{U}V_{U}^{S^{\prime }}}{\mu_{U}\left(1-\varepsilon \right)}\right]}^{2}+\pi_{L}f\\ +{V_{L}^{S}}^{\prime }\left[\alpha_{L}E_{L}+\frac{\alpha_{U}\left(\pi_{U}\gamma \beta_{U}+\alpha_{U}V_{U}^{S^{\prime }}\right)}{\mu_{U}\left(1-\varepsilon \right)}-\delta R\right] \end{array}\right\}\text{.} \end{array}$$

To solve *ε*, the first derivative of the function on the right of the formula (51) is taken and set equal to zero, which gives:

(52) $$\begin{array}{c} \varepsilon =\left\{\begin{array}{c} \frac{2\left(\omega +\alpha_{U}V_{L}^{S^{\prime }}+\gamma \beta_{U}\pi_{L}\right)-\left(\gamma \beta_{U}\pi_{U}+\alpha_{U}V_{U}^{S^{\prime }}\right)}{2\left(\omega +\alpha_{U}V_{L}^{S^{\prime }}+\gamma \beta_{U}\pi_{L}\right)+\left(\gamma \beta_{U}\pi_{U}+\alpha_{U}V_{U}^{S^{\prime }}\right)},B>C,\\ 0,B<C\text{,} \end{array}\right. \end{array}$$

With *B*=2(*ω*+*α*_*U*_*V*_*L*_^*S*′^+*γβ*_*U*_*π*_*L*_), *C*=*γβ*_*U*_*π*_*U*_+*α*_*U*_*V*_*U*_^*S*′^.

Inputting equations (48) and (50) into equation (47), it is concluded that:

(53) $$\begin{array}{c} \begin{array}{c} \rho V_{U}^{S}\left(R\right)=\left(\lambda \pi_{U}\gamma -\delta V_{U}^{S^{\prime }}\right)R-\frac{{\left(\alpha_{U}V_{U}^{S^{\prime }}+\beta_{U}\pi_{U}\gamma \right)}^{2}}{2\mu_{U}{\left(1-\varepsilon \right)}^{2}}+\pi_{U}f+\frac{\varepsilon \left(\alpha_{U}V_{U}^{S^{\prime }}+\beta_{U}\pi_{U}\gamma \right)}{2\mu_{U}{\left(1-\varepsilon \right)}^{2}}\\ +V_{U}^{S^{\prime }}\left[\frac{\alpha_{L}\left(\alpha_{L}{V_{L}^{S}}^{\prime }+\beta_{L}\pi_{L}\gamma \right)}{\mu_{L}}+\frac{\alpha_{U}\left(\alpha_{U}V_{U}^{S^{\prime }}+\beta_{U}\pi_{U}\gamma \right)}{\mu_{U}\left(1-\varepsilon \right)}\right]\\ +\pi_{U}\gamma \left[\frac{\beta_{L}\left(\alpha_{L}{V_{L}^{S}}^{\prime }+\beta_{L}\pi_{L}\gamma \right)}{\mu_{L}}+\frac{\beta_{U}\left(\alpha_{U}V_{U}^{S^{\prime }}+\beta_{U}\pi_{U}\gamma \right)}{\mu_{U}\left(1-\varepsilon \right)}\right] \end{array}\text{.} \end{array}$$

Inputting equations (48) and (50) into equation (49), it is concluded that:

(54) $$\begin{array}{c} \begin{array}{c} \rho V_{L}^{S}\left(R\right)=\left(\lambda \pi_{L}\gamma -\delta {V_{L}^{S}}^{\prime }\right)R+\pi_{L}f-\frac{{\left(\alpha_{L}V_{U}^{S^{\prime }}+\beta_{L}\pi_{L}\gamma \right)}^{2}}{2\mu_{L}}\\ +{V_{L}^{S}}^{\prime }\left[\frac{\alpha_{L}\left(\alpha_{L}V_{U}^{S^{\prime }}+\beta_{L}\pi_{L}\gamma \right)}{\mu_{L}}+\frac{\alpha_{U}\left(\alpha_{U}V_{U}^{S^{\prime }}+\beta_{U}\pi_{U}\gamma \right)}{\mu_{U}\left(1-\varepsilon \right)}\right]\\ +\frac{\omega \left(\alpha_{U}V_{U}^{S^{\prime }}+\beta_{U}\pi_{U}\gamma \right)}{\mu_{U}\left(1-\varepsilon \right)}-\frac{\varepsilon {\left(\alpha_{U}V_{U}^{S^{\prime }}+\beta_{U}\pi_{U}\gamma \right)}^{2}}{2\mu_{U}{\left(1-\varepsilon \right)}^{2}}\\ +\pi_{L}\gamma \left[\frac{\beta_{L}\left(\alpha_{L}V_{U}^{S^{\prime }}+\beta_{L}\pi_{L}\gamma \right)}{\mu_{L}}+\frac{\beta_{U}\left(\alpha_{U}V_{U}^{S^{\prime }}+\beta_{U}\pi_{U}\gamma \right)}{\mu_{U}\left(1-\varepsilon \right)}\right] \end{array}\text{.} \end{array}$$

From the structural characteristics of equations (53) and (54), the linear optimal value function for *R* is the solution to the HJB equation. Therefore, it is assumed that *V*_*L*_^*S*^(*R*)'s and *V*_*U*_^*S*^(*R*)'s linear analytic equations for *R* are:

(55) $$\begin{array}{c} V_{L}^{S}\left(R\right)=g_{1}R+g_{2}\text{,} \end{array}$$

(56) $$\begin{array}{c} V_{U}^{S}\left(R\right)=h_{1}R+h_{2}\text{.} \end{array}$$

With g_1_, g_2_, *h*_1_, and *h*_2_ being undetermined coefficients. From equations (55) and (56), it is further determined that:

(57) $$\begin{array}{c} V_{U}^{S^{\prime }}\left(R\right)=g_{1}\text{,} \end{array}$$

(58) $$\begin{array}{c} V_{U}^{S^{\prime }}\left(R\right)=h_{1}\text{.} \end{array}$$

Equations (55)–(58) are inputted into equations (53) and (54). With the undetermined coefficients method, the values of g_1_, g_2_, *h*_1_, and *h*_2_ could be obtained:

(59) $$\begin{array}{c} g_{1}=\frac{\lambda \pi_{L}\gamma }{\delta +\rho }\text{,} \end{array}$$

(60) $$\begin{array}{c} \begin{array}{c} g_{2}=\frac{\pi_{L}f}{\rho }+\frac{\pi_{L}\gamma^{2}\pi_{U}{\left[\lambda \alpha_{U}+\left(\delta +\rho \right)\beta_{U}\right]}^{2}}{\rho \left(1-\varepsilon \right){\left(\delta +\rho \right)}^{2}\mu_{U}}+\frac{\gamma^{2}\pi_{L}^{2}{\left[\lambda \alpha_{L}+\left(\delta +\rho \right)\beta_{L}\right]}^{2}}{2\rho {\left(\delta +\rho \right)}^{2}{µ}_{L}}\\ +\frac{\omega \gamma \pi_{U}\left[\lambda \alpha_{U}+\left(\delta +\rho \right)\beta_{U}\right]}{\rho \left(1-\varepsilon \right)\left(\delta +\rho \right)\mu_{U}}-\frac{\gamma^{2}\pi_{U}^{2}\varepsilon {\left[\lambda \alpha_{U}+\left(\delta +\rho \right)\beta_{U}\right]}^{2}}{2\rho {\left(1-\varepsilon \right)}^{2}{\left(\delta +\rho \right)}^{2}\mu_{U}} \end{array}\text{,} \end{array}$$

(61) $$\begin{array}{c} h_{1}=\frac{\lambda \pi_{U}\gamma }{\delta +\rho }\text{,} \end{array}$$

(62) $$\begin{array}{c} h_{2}=\frac{\pi_{U}f}{\rho }+\frac{\pi_{U}^{2}\gamma^{2}{\left[\lambda \alpha_{U}+\left(\delta +\rho \right)\beta_{U}\right]}^{2}}{2\rho \left(1-\varepsilon \right){\left(\delta +\rho \right)}^{2}\mu_{U}}+\frac{\pi_{U}\gamma^{2}\pi_{L}{\left[\lambda \alpha_{L}+\left(\delta +\rho \right)\beta_{L}\right]}^{2}}{\rho {\left(\delta +\rho \right)}^{2}{µ}_{L}}\text{.} \end{array}$$

Inputting equations (57), (58), (59), and (61) into equation (52), can get the equation (40) of cost-subsidy proportion of opinion leaders.

Inputting equation (40) into equations (60) and (62), the values of g_2_ and *h*_2_ under *γ*(*π*_*U*_ − 2*π*_*L*_)[*λα*_*U*_ + (*δ* + *ρ*)*β*_*U*_] < 2(*δ* + *ρ*)*ω* could be obtained:

(63) $$\begin{array}{c} \begin{array}{c} +\frac{{\left[\alpha_{L}\lambda +\beta_{L}\left(\delta +\rho \right)\right]}^{2}\pi_{L}^{2}\gamma^{2}}{2\rho {\left(\delta +\rho \right)}^{2}{µ}_{L}}+\frac{\pi_{L}f}{\rho }+\frac{\lambda \gamma \alpha_{U}\left(2\pi_{L}+\pi_{U}\right)\left[2\omega +\gamma \beta_{U}\left(2\pi_{L}+\pi_{U}\right)\right]}{4\rho \left(\delta +\rho \right)\mu_{U}} \end{array}\text{,} \end{array}$$

(64) $$\begin{array}{c} h_{2}=\frac{\pi_{U}\beta_{U}^{2}\left(2\pi_{L}+\pi_{U}\right)\gamma^{2}}{4\rho \mu_{U}}+\frac{{\left[\alpha_{L}\lambda +\beta_{L}\left(\delta +\rho \right)\right]}^{2}\pi_{U}\pi_{L}\gamma^{2}}{\rho {\left(\delta +\rho \right)}^{2}{µ}_{L}}+\frac{\pi_{U}\alpha_{U}^{2}\lambda^{2}\gamma^{2}\left(2\pi_{L}+\pi_{U}\right)}{4\rho {\left(\delta +\rho \right)}^{2}\mu_{U}} \end{array}$$

Inputting equations (57) and (59) into equation (50), the optimal equilibrium strategy for opinion leaders is shown as equation (41); inputting equations (40), (58), and (61) into equation (48), the optimal equilibrium strategy for Internet users is shown as equation (42); inputting equations (41) and (42) into equation (2), the optimal trajectory of total volume of real information and the steady-state value are shown as equation (43); inputting equations (59), (60), and (63) into equation (55), the optimal benefit of opinion leaders is shown as equation (44); inputting equations (61), (62), and (64) into equation (56), the optimal benefit of Internet users is shown as equation (45); from equations (44) and (45), the optimal benefit for the whole false information clarification system is shown as equation (46).

So far, Theorem 3 has been verified.

**Deduction 3 .**

From Theorem 3, it is known that opinion leaders would provide a cost subsidy to Internet users when *γ*(*π*_*U*_ − 2*π*_*L*_)[*λα*_*U*_+(*δ*+*ρ*)*β*_*U*_]/2(*δ*+*ρ*)*ω* < 1. In addition, the proportion of the cost subsidy provided by opinion leaders is affected simultaneously by the attention level of major emergencies, direct benefit per unit opinion leaders gained from effort paid by Internet users, marginal traffic benefit for opinion leaders and Internet users, the total volume of real information, and influential coefficients of the total volume of real information, and the effort level of Internet users on social platforms.

Under decentralized decision-making with a cost subsidy provided by opinion leaders, the optimal equilibrium strategy of opinion leaders (i.e., their effort level) is consistent with that under decentralized decision-making without a cost subsidy; the optimal equilibrium strategy (i.e., their effort level) is additionally affected by the cost-subsidy proportion of opinion leaders. The optimal trajectory of the total volume of real information is dependent on efforts paid by opinion leaders and Internet users, while the optimal benefit of the false information clarification system increases with the growth of the total volume of real information. This means that the more effort paid by opinion leaders and Internet users, the more benefit they will receive. Details of the interrelated relationship are shown in Table 3.

## 4. Model Analysis

### 4.1. Comparative Analysis

From equation (40), it is known that when *γ*(*π*_*U*_ − 2*π*_*L*_)[*λα*_*U*_ + (*δ* + *ρ*)*β*_*U*_] < 2(*δ* + *ρ*)*ω*, opinion leaders will provide a cost subsidy for Internet users; when *γ*(*π*_*U*_ − 2*π*_*L*_)[*λα*_*U*_ + (*δ* + *ρ*)*β*_*U*_] ≥ 2(*δ* + *ρ*)*ω*, *ε* = 0, this means the cost-subsidy proportion is zero, so Internet users get zero cost subsidy, and the decision behavior at this time is the same as that under decentralized decision-making. Therefore, this study will do a comparative analysis under the circumstance of *γ*(*π*_*U*_ − 2*π*_*L*_)[*λα*_*U*_ + (*δ* + *ρ*)*β*_*U*_] < 2(*δ* + *ρ*)*ω* and determine the optimal equilibrium strategy for opinion leaders and Internet users, the steady-state value of the total volume of real information, and the optimal benefit for opinion leaders, Internet users, and the system under three decision behaviors (i.e., centralized decision-making, decentralized decision-making, and cost-subsidy decision-making). Deductions are as follows:

**Deduction 4 .**

The optimal strategy of opinion leaders under different decision behaviors: *E*_*L*_^*N*^*∗*^^=*E*_*L*_^*S*^*∗*^^ < *E*_*L*_^*C*^*∗*^^

The optimal strategy of Internet users under different decision behaviors: *E*_*U*_^*N*^*∗*^^ < *E*_*U*_^*S*^*∗*^^ < *E*_*U*_^*C*^*∗*^^

**Verification 4.**

Comparing equations (6), (27), and (41):

(65) $$\begin{array}{c} E_{L}^{N^{*}}-E_{L}^{S^{*}}=0E_{L}^{C^{*}}-E_{L}^{S^{*}}=\frac{\gamma \pi_{U}\left[\lambda \alpha_{L}+\left(\delta +\rho \right)\beta_{L}\right]}{\mu_{L}\left(\delta +\rho \right)}\text{,} \end{array}$$

Because all related parameters exceed zero, *E*_*L*_^*N*^*∗*^^ = *E*_*L*_^*S*^*∗*^^ < *E*_*L*_^*C*^*∗*^^ is met;

Comparing equations (7), (28), and (42):

(66) $$\begin{array}{c} \begin{array}{c} E_{U}^{S^{*}}-E_{U}^{N^{*}}=\frac{2\left(\delta +\rho \right)\omega +\gamma \left(2\pi_{L}-\pi_{U}\right)\left[\lambda \alpha_{U}+\left(\delta +\rho \right)\beta_{U}\right]}{2\left(\delta +\rho \right)\mu_{U}}\\ E_{U}^{C^{*}}-E_{U}^{S^{*}}=\frac{\gamma \pi_{U}\left[\lambda \alpha_{U}+\left(\delta +\rho \right)\beta_{U}\right]}{2\mu_{U}\left(\delta +\rho \right)} \end{array}\text{.} \end{array}$$

Because all related parameters exceed zero, *E*_*U*_^*N*^*∗*^^ < *E*_*U*_^*S*^*∗*^^ < *E*_*U*_^*C*^*∗*^^ is met; So far,Deduction 4 has been verified.

From Deduction 4, it is known that whatever the value of parameters, the optimal effort level paid by opinion leaders and Internet users under centralized decision-making behavior is higher than that under the other two decision-making behaviors. Under decentralized decision-making and cost-subsidy decision-making, the optimal effort paid by opinion leaders is the same; only when a certain condition is met between the attention level of a major emergency and the direct benefit per unit opinion leaders gained from the effort paid by Internet users, marginal traffic benefit for opinion leaders and Internet users, total volume of real information, and the influential coefficients of effort level of Internet users on social platform traffic will the optimal effort level paid by Internet users under decentralized decision-making be lower than that under cost-subsidy decision-making. Otherwise, the results are the same.

**Deduction 5 .**

Steady-state value of the total volume of real information under different circumstances: *R*_*S*_^*N*^ < *R*_*S*_^*S*^ < *R*_*S*_^*C*^.

**Verification 5.**

By comparing the steady-state value of the total volume of real information, it is known that:

(67) $$\begin{array}{c} \begin{array}{c} R_{S}^{C}-R_{S}^{S}=\frac{\gamma \alpha_{L}\pi_{U}\left[\lambda \alpha_{L}+\left(\delta +\rho \right)\beta_{L}\right]}{\delta \left(\delta +\rho \right)\mu_{L}}+\frac{\gamma \alpha_{U}\pi_{U}\left[\lambda \alpha_{U}+\left(\delta +\rho \right)\beta_{U}\right]}{2\delta \mu_{U}\left(\delta +\rho \right)}\\ R_{S}^{S}-R_{S}^{N}=\frac{2\alpha_{U}\left(\delta +\rho \right)\omega +\gamma \alpha_{U}\left(2\pi_{L}-\pi_{U}\right)\left[\lambda \alpha_{U}+\left(\delta +\rho \right)\beta_{U}\right]}{2\delta \left(\delta +\rho \right)\mu_{U}} \end{array}\text{.} \end{array}$$

Because all related parameters exceed zero and *γ*(*π*_*U*_ − 2*π*_*L*_)[*λα*_*U*_+(*δ*+*ρ*)*β*_*U*_] < 2(*δ*+*ρ*)*ω*, *R*_*S*_^*N*^ < *R*_*S*_^*S*^ < *R*_*S*_^*C*^ is met.

So far,Deduction 5 has been verified.

From Deduction 5, it is known that whatever the value of parameters, the steady-state value of the total volume of real information under centralized decision-making behavior is greater than that of under the other two decision-making behavior. Only when a certain condition is met between the attention level of a major emergency, the direct benefit per unit opinion leaders gained from effort paid by Internet users, marginal traffic benefit for opinion leaders and Internet users, the total volume of real information, and the influential coefficients of effort level of Internet users on social platform traffic will the steady-state value of the total volume of real information under decentralized decision-making behavior be smaller than that under cost-subsidy decision-making behavior. Otherwise, the results are the same.

**Deduction 6 .**

The optimal benefit for opinion leaders under different circumstances is: *V*_*L*_^*N*^*∗*^^(*R*) < *V*_*L*_^*S*^*∗*^^(*R*).

The optimal benefit for Internet users under different circumstances is: *V*_*U*_^*N*^*∗*^^(*R*) < *V*_*U*_^*S*^*∗*^^(*R*).

The optimal benefit for the system under different circumstances is: *V*^*N*^*∗*^^(*R*) < *V*^*S*^*∗*^^(*R*) < *V*^*C*^*∗*^^(*R*)

**Verification 6.**

Comparing (9) and (44):

(68) $$\begin{array}{c} V_{L}^{S^{*}}\left(R\right)-V_{L}^{N^{*}}\left(R\right)=\frac{\lambda \pi_{L}\gamma }{\delta +\rho }\left(R_{S}^{S}-R_{S}^{N}\right)\\ +\frac{{\left\{2\left(\delta +\rho \right)\omega +\gamma \left(2\pi_{L}-\pi_{U}\right)\left[\lambda \alpha_{U}+\left(\delta +\rho \right)\beta_{U}\right]\right\}}^{2}}{8\rho {\left(\delta +\rho \right)}^{2}\mu_{U}}\text{.} \end{array}$$

From Deduction 5 it is known that *R*_*S*_^*N*^ < *R*_*S*_^*S*^, all related parameters exceed zero and *γ*(*π*_*U*_ − 2*π*_*L*_)[*λα*_*U*_ + (*δ* + *ρ*)*β*_*U*_] < 2(*δ* + *ρ*)*ω*; therefore, *V*_*L*_^*N*^*∗*^^(*R*) < *V*_*L*_^*S*^*∗*^^(*R*) is met.

Comparing (10) and (45):

(69) $$\begin{array}{c} V_{U}^{S^{*}}\left(R\right)-V_{U}^{N^{*}}\left(R\right)=\frac{\lambda \pi_{U}\gamma }{\delta +\rho }\left(R_{S}^{S}-R_{S}^{N}\right)+\frac{\varepsilon \pi_{U}^{2}\gamma^{2}{\left[\lambda \alpha_{U}+\left(\delta +\rho \right)\beta_{U}\right]}^{2}}{2\rho \left(1-\varepsilon \right){\left(\delta +\rho \right)}^{2}\mu_{U}}\text{.} \end{array}$$

From Deduction 5, it is known that *R*_*S*_^*N*^ < *R*_*S*_^*S*^, all related parameters exceed zero, and 0 ≤ *ε* < 1; therefore, *V*_*U*_^*N*^*∗*^^(*R*) < *V*_*U*_^*S*^*∗*^^(*R*) is met.

Because *V*^*N*^*∗*^^(*R*) = *V*_*L*_^*N*^*∗*^^(*R*) + *V*_*U*_^*N*^*∗*^^(*R*), *V*^*S*^*∗*^^(*R*) = *V*_*L*_^*S*^*∗*^^(*R*) + *V*_*U*_^*S*^*∗*^^(*R*) and *V*_*L*_^*N*^*∗*^^(*R*) < *V*_*L*_^*S*^*∗*^^(*R*), *V*_*U*_^*N*^*∗*^^(*R*) < *V*_*U*_^*S*^*∗*^^(*R*), *V*_*L*_^*N*^*∗*^^(*R*) < *V*_*L*_^*S*^*∗*^^(*R*), *V*_*U*_^*N*^*∗*^^(*R*) < *V*_*U*_^*S*^*∗*^^(*R*) must exist. Therefore, only comparing the value of *V*^*C*^*∗*^^(*R*) and *V*^*S*^*∗*^^(*R*) is needed. Comparing (30) and (46):

(70) $$\begin{array}{c} V^{C^{*}}\left(R\right)-V^{S^{*}}\left(R\right)=\frac{\lambda \gamma \left(\pi_{L}+\pi_{U}\right)}{\delta +\rho }\left(R_{S}^{C}-R_{S}^{S}\right)\\ +\frac{\gamma^{2}\pi_{U}^{2}\left\{4{\left[\lambda \alpha_{L}+\left(\delta +\rho \right)\beta_{L}\right]}^{2}\mu_{U}+{\left[\lambda \alpha_{U}+\left(\delta +\rho \right)\beta_{U}\right]}^{2}{µ}_{L}\right\}}{8\rho {\left(\delta +\rho \right)}^{2}\mu_{U}{µ}_{L}}\text{.} \end{array}$$

From Deduction 5, it is known that *R*_*S*_^*S*^ < *R*_*S*_^*C*^, and all related parameters exceed zero; therefore, *V*^*N*^*∗*^^(*R*) < *V*^*S*^*∗*^^(*R*) < *V*^*C*^*∗*^^(*R*) is met.

So far, Deduction 6 has been verified.

From Deduction 6, it is known that the optimal benefit for both opinion leaders and Internet users under cost-subsidy decision-making is greater than that under decentralized decision-making. For the whole system, the optimal benefit obtained under centralized decision-making, cost-subsidy decision-making, and decentralized decision-making diminishes.

### 4.2. Numerical Simulation Analysis

To further verify the above theoretical analysis, MATLAB 2017 has been applied to assign theoretical parameters under decentralized, centralized, and cost-subsidy decision-making of opinion leaders and Internet users, so as to explore the progressive evolution of the total volume of real information, the benefit of opinion leaders and Internet users, and the total benefit of false information clarification system over time in a more visual way. Because relevant parameters of opinion leaders and Internet users could not be obtained directly in the real world, this study will try its best to rationalize the above values according to the real situation. The assignment of relevant parameters is as follows:

(71) $$\begin{array}{c} \left\{\begin{array}{c} \mu_{L}=6,\mu_{U}=4.5,\alpha_{L}=3,\alpha_{U}=2,\delta =1\text{,}\\ R_{0}=0,f=1,\gamma =0.7,\beta_{L}=1.5,\beta_{U}=1\text{,}\\ \rho =0.2,\pi_{L}=4,\pi_{U}=2,\omega =1.5,\lambda =0.8\text{.} \end{array}\right. \end{array}$$

Based on the above assignments, the equilibrium results of differential games under different decision-making behaviors are given in Table 4.

The equilibrium results in Table 4 prove that the theoretical analysis in Deduction 4–Deduction 6 is correct.

#### 4.2.1. Simulation Analysis of Changing Trajectory of the Total Volume of Real Information

**Figure 1:**
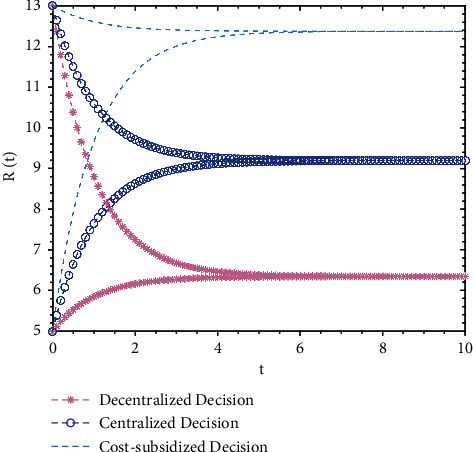
The evolution trajectory of the total volume of real information under three decisions.

Keeping the values of other parameters unchanged, *R*_0_ = 5 and *R*_0_ = 13 are taken as the total volume of real information at the initial moment, and a numerical simulation analysis is applied to the optimal trajectory of the total volume of real information under three different decision-making types. The evolution trajectory of the total volume of real information over time is shown in Figure 1.

From Figure 1, it is found that the evolution trajectories of the total volume of real information under the three decision-making types are related to the initial value of the total volume of real information. When the initial value is relatively high, the total volume of real information diminishes over time; when the initial value is relatively low, the total volume of real information increases over time. Both converge to the same steady-state value. From the deductions above, it is known that the steady-state value of the total volume of real information is irrelevant to the initial value, but relevant only to different decision-making behaviors.

Because the cost-subsidy behavior of opinion leaders under subsidized decision-making behavior could reach Pareto improvement on the total volume of real information, the steady-state value of the total volume of real information under decentralized decision-making is the smallest, followed by that under cost-subsidy decision-making, and the value under centralized decision reaches Pareto optimality.

#### 4.2.2. Simulation Analysis of Changing Trajectories of the Total Benefit of Opinion Leaders and Internet Users

**Figure 2:**
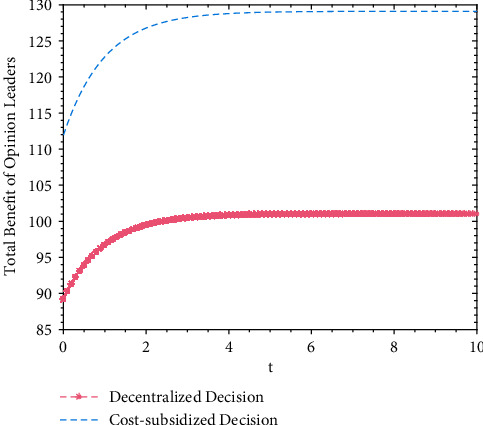
Trajectories of opinion leaders' total benefit under decentralized and cost-subsidy decision.

**Figure 3:**
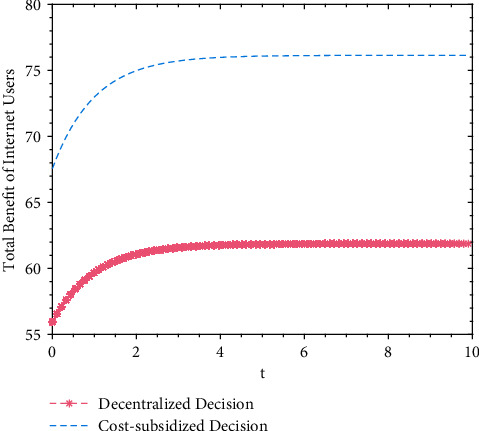
Trajectories of internet users' total benefit under decentralized and cost-subsidy decision.

A numerical simulation analysis is applied to the benefits of opinion leaders under decentralized and cost-subsidy decision-making, and the trajectories of opinion leaders' total benefit over time are shown in Figure 2. A numerical simulation analysis is also applied to the benefits of Internet users under decentralized and cost-subsidy decision-making, and the trajectories of Internet users' total benefits over time are shown in Figure 3.

Based on Figures 2 and 3, it is found that the total benefit of opinion leaders and Internet users increases slightly over time, and finally plateaus at a fixed value. After reaching the fixed value (the maximum), the total benefits of both parties will not change over time, and both parties' total benefits are higher under cost-subsidy decision-making than under decentralized decision. Although additional cost subsidy for Internet users is needed during cost-subsidy decision-making, the total benefits are significantly improved. This is because the total volume of real information achieves Pareto improvement; with the increase of the total volume of real information, the benefit for opinion leaders and Internet users increases.

#### 4.2.3. Simulation Analysis of the Trajectory of the System's Total Benefit

**Figure 4:**
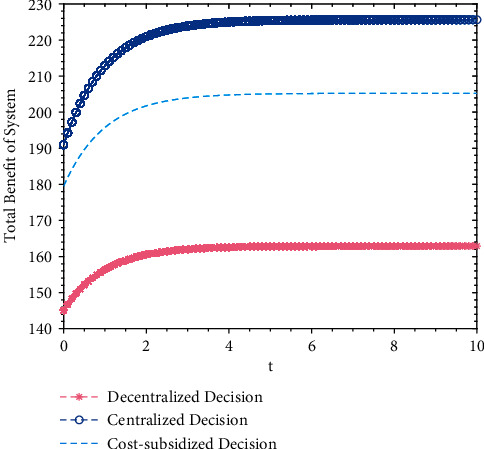
Trajectories of the system's total benefit over time under three decision behavior.

A numerical simulation analysis is applied to the total benefit of the system under decentralized, centralized, and cost-subsidy decision-making, and the trajectories of the system's total benefit over time are shown in Figure 4.

Based on Figure 4, it is found that regardless of the decision-making behavior, the system's total benefit increased. Trajectories under the three decision-making behaviors plateaued at a fixed value over time, which means total benefit will not increase with time when it reaches the maximum. Under centralized decision-making, because both opinion leaders and Internet users set the maximum of the system's benefit as their goals, they fully cooperate with each other without changing their own behavior out of personal interest; therefore, the system's total benefit reaches Pareto optimality, higher than that under decentralized or cost-subsidy decision-making; under decentralized decision-making, because both opinion leaders and Internet users set the maximum of their own benefit as their goals, they will not change their behavior to improve the system's benefit, making the system's benefit the lowest among all three decision-making types; under cost-subsidy decision-making, which is an improvement to decentralized decision-making, opinion leaders provide a cost subsidy to Internet users so as to achieve Pareto improvement for the system's total benefit. Therefore, the system's total benefit under cost-subsidy decision-making is higher than that under decentralized decision-making but lower than that under centralized decision-making due to the failure to achieve Pareto optimality.

#### 4.2.4. Analysis of Each Parameter's Sensibility to Equilibrium Results

**Figure 5:**
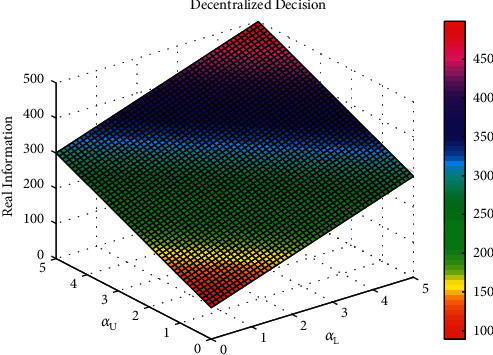
The influence of parameter *α*_L_ and *α*_U_ on the total volume of real information under decentralized decision.

Analyses of each important parameter's sensibility to equilibrium results under decentralized, centralized, and cost-subsidy decision-making are carried out. Limited by the length of this study, only several parameters' sensibility analysis figures are listed below, with the rest of the parameters' sensibilities analyzed based on −20%, −10%, +10%, and +20% of the previously mentioned (71) standard value. Details of the changes of equilibrium results with the increase of parameter value are given in Figure 5.

**Figure 6:**
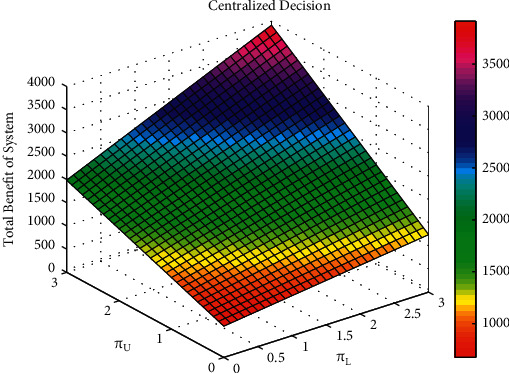
The influence of parameter *π*_L_ and *π*_U_ on the system's total benefit under centralized decision.

**Figure 7:**
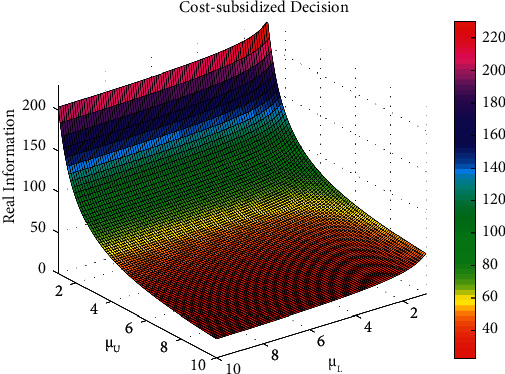
The influence of parameter *μ*_L_ and *μ*_U_ on the total volume of real information under cost-subsidy decision.

The influence of parameters *α*_L_ and *α*_U_ on the total volume of real information under decentralized decision-making, that of parameters *π*_L_ and *π*_U_ on the system's total benefit under centralized decision-making, and that parameters *μ*_L_ and *μ*_U_ on the total volume of real information under cost-subsidy decision-making are shown in Figures 5–7, respectively.

Based on Figure 5, the greater the influential coefficient of effort level of opinion leaders and Internet users on the total volume of real information under decentralized decision-making, the more real information on social platforms. Based on Figure 6, opinion leaders' and Internet users' marginal traffic under centralized decision-making is positively related to the system's total benefit, which means the greater the marginal traffic, the higher the system's total benefit. Based on Figure 7, opinion leaders' and Internet users' effort cost coefficient is negatively related to the total volume of real information under cost-subsidy decision-making, which means the greater the effort cost coefficient, the less real information on social platforms.

From the results in Table 5, it is found that:

1. Regardless of decision behavior, opinion leaders and Internet users marginal traffic benefit *π*_L_ and *π*_U_, effort level's influential coefficients *α*_L_ and *α*_U_ on the total volume of real information, effort level's influential coefficients *β*_L_ and *β*_U_ on the traffic of social platform, attention level *γ* received by major emergencies, and the total volume of real information's influential coefficient *λ* on the traffic of social platform are positively related to the total volume of real information and the system's total benefit.
2. Regardless of decision behavior, the effort cost coefficients *μ*_L_ and *μ*_U_ of opinion leaders and Internet users, the natural dissipation coefficient of real information, and the discount rate *ρ* are negatively correlated with the total volume of real information and the system's total benefit.
3. A social platform's initial traffic has no influence on the total volume of real information, but it is positively correlated with the system's total benefit. Under decentralized decision-making, the direct benefit per unit obtained by opinion leaders from the effort of Internet users *ω* has no influence on the total volume of real information, but it is positively correlated with the system's total benefit; under centralized and cost-subsidy decision-making, the direct benefit per unit obtained by opinion leaders from the effort of Internet users *ω* is positively correlated with the total volume of real information and the system's total benefit.

## 5. Conclusion

Under the background of false information on social platforms after major emergencies, this study explores the dynamic optimization of a clarification system consisting of false information released by opinion leaders and Internet users. Based on optimal control theory and differential game theory, differential game models under decentralized, centralized, and cost-subsidy decision-making are constructed, and opinion leaders' and Internet users' optimal equilibrium strategies and optimal benefit, optimal trajectory, and steady-state value of the total volume of real information, and the optimal benefit of the false information clarification system are obtained. The following conclusions can be drawn after comparative analyses and numerical simulations:

1. Compared with the other two decision behaviors, under centralized decision-making, opinion leaders' and Internet users' optimal equilibrium strategies and optimal benefit, the optimal trajectory and steady-state value of the total volume of real information, and the optimal benefit of false information clarification system reach their maxima; therefore, Pareto optimality is achieved. This means that centralized decision-making could, to the largest extent, reduce public panic caused by false information after major emergencies, and could be regarded as the optimal decision-making type for opinion leaders and Internet users.
2. Cost-subsidy decision-making, as compared with centralized decision-making, fails to achieve Pareto optimality but efficiently improves decentralized behavior because opinion leaders provide a cost subsidy for Internet users. When the cost subsidy reaches a certain proportion, the optimal benefit of opinion leaders and Internet users, the optimal trajectory of the total volume of real information and its steady-state value, and the false information clarification system could achieve Pareto improvement. Although cost-subsidy decision-making fails to achieve Pareto optimality, as opinion leaders and Internet users could not be absolutely rational to reach centralized decision, cost-subsidy decision-making has its practical significance.
3. Opinion leaders' and Internet users' effort cost coefficient, discount rate, and real information's natural dissipation rate are negatively correlated with the volume of real information. Therefore, in the real world, a certain extent of subsidy could be provided by social platforms or the relevant departments of the government to opinion leaders and Internet users, so as to increase the volume of real information and reduce public panic.

## Figures and Tables

**Table 1:** Influence of different parameters on optimal equilibrium strategy of opinion leaders and internet users under decentralized decision strategy.

	*γ*	*π*_L_	*π*_U_	*λ*	*α*_L_	*α*_U_	*β*_L_	*β*_U_	*μ*_L_	*μ*_U_
*E*_*L*_^*N*^	↗	↗	—	↗	↗	—	↗	—	↘	—
*E*_*U*_^*N*^	↗	—	↗	↗	—	↗	—	↗	—	↘

*Note.* ↗ refers to positive influence, ↘ refers to negative influence, —refers to irrelevance.

**Table 2:** Influence of different parameters on optimal equilibrium strategy of opinion leaders and internet users under centralized decision strategy.

	*γ*	*π*_L_	*π*_U_	*λ*	*α*_L_	*α*_U_	*β*_L_	*β*_U_	*μ*_L_	*μ*_U_	*ω*
*E*_*L*_^*C*^	↗	↗	↗	↗	↗	—	↗	—	↘	—	—
*E*_*U*_^*C*^	↗	↗	↗	↗	—	↗	—	↗	—	↘	↗

*Note.* ↗ refers to positive influence, ↘ refers to negative influence, —refers to irrelevance.

**Table 3:** Influence of different parameters on optimal equilibrium strategy of opinion leaders and internet users and cost-subsidy proportion under decentralized decision with cost subsidy.

	*γ*	*π*_L_	*π*_U_	*λ*	*α*_L_	*α*_U_	*β*_L_	*β*_U_	*μ*_L_	*μ*_U_	*ω*
*E*_*L*_^*S*^	↗	↗	—	↗	↗	—	↗	—	↘	—	—
*E*_*U*_^*S*^	↗	↗	↗	↗	—	↗	—	↗	—	↘	↗
Ε	↗	↗	↘	↗	—	↗	—	↗	—	—	↗

*Note.* ↗ refers to positive influence, ↘ refers to negative influence, —refers to irrelevance.

**Table 4:** Equilibrium results of differential game under different decision behavior.

	Decentralized decision	Centralized decision	Cost-subsidy decision
Effort level of opinion leaders	1.6333	2.4500	1.6333
Effort level of internet users	0.7259	2.5111	2.1481
Cost-subsidy proportion	—	—	0.6621
Total volume of real information	6.3519	12.3722	9.1963
Total benefit of opinion leaders	101.0315	—	129.0967
Total benefit of internet users	61.8735	—	76.1431
Total benefit of the system	162.9049	225.6186	205.2398

*Note.* 4 digits after the decimal point kept.

**Table 5:** Analysis of key parameters' sensitivity to differential game equilibrium results.

Key parameters	Decentralized decision		Centralized decision		Cost-subsidy decision	
	Total volume of real information	System's total benefit	Total volume of real information	System's total benefit	Total volume of real information	System's total benefit
*λ*↑	↑	↑	↑	↑	↑	↑
*ω*↑	—	↑	↑	↑	↑	↑
*γ*↑	↑	↑	↑	↑	↑	↑
f↑	—	↑	—	↑	—	↑
*μ*_L_↑	↓	↓	↓	↓	↓	↓
*μ*_U_↑	↓	↓	↓	↓	↓	↓
*π*_L_↑	↑	↑	↑	↑	↑	↑
*π*_U_↑	↑	↑	↑	↑	↑	↑
*α*_L_↑	↑	↑	↑	↑	↑	↑
*α*_U_↑	↑	↑	↑	↑	↑	↑
*β*_L_↑	↑	↑	↑	↑	↑	↑
*β*_U_↑	↑	↑	↑	↑	↑	↑
*δ*↑	↓	↓	↓	↓	↓	↓
*ρ*↑	↓	↓	↓	↓	↓	↓

Note: ↗ refers to positive relevance, ↘ refers to negative relevance, —refers to irrelevance.

## Data Availability

The authors confirm that the data supporting the findings of this study are included within the article.
